# Surface Evaluation of a Novel Acid-Etching Solution for Zirconia and Lithium Disilicate

**DOI:** 10.3390/ma18122912

**Published:** 2025-06-19

**Authors:** Clint Conner, Fabio Andretti, Alfredo I. Hernandez, Silvia Rojas-Rueda, Francisco X. Azpiazu-Flores, Brian R. Morrow, Franklin Garcia-Godoy, Carlos A. Jurado, Abdulrahman Alshabib

**Affiliations:** 1 Division of Oper Dent, Department of General Dentistry, College of Dentistry, University of Tennessee Health Science Center, Memphis, TN 38104, USA; 2 Prosthodontics Department, Arizona School of Dentistry and Oral Health, A.T. Still University, Mesa, AZ 85206, USA; 3 Division of Dental Biomaterials, Department of Clinical and Community Sciences, School of Dentistry, University of Alabama at Birmingham, Birmingham, AL 35233, USA; 4 Division of Restorative and Prosthetic Dentistry, College of Dentistry, Ohio State University, Columbus, OH 43210, USA; 5 Department of Bioscience Research, College of Dentistry, University of Tennessee Health Science Center, Memphis, TN 382014, USA; 6 School of Dental Medicine, Ponce Health Sciences University, Ponce, PR 00732, USA; 7 Department of Restorative Dentistry, King Saud University, Riyadh 11545, Saudi Arabia

**Keywords:** acid etching, surface evaluation, roughness, zirconia, lithium disilicate

## Abstract

The current investigation evaluated a novel acid-etching solution containing hydrochloric acid (HCl), hydrofluoric acid (HF), nitric acid (HNO_3_), orthophosphoric acid (H_3_PO_4_), and sulfuric acid (H_2_SO_4_) designed for etching zirconia ceramics. Achieving reliable bonding to zirconia is challenging due to its chemical inertia, unlike lithium disilicate, which can be effectively conditioned with HF etching. One hundred and twenty specimens of zirconia and lithium disilicate underwent etching with the experimental solution for six different durations: control, 20 s, 60 s, 5 min, 30 min, and 1 h. Surface roughness was assessed using 3D optical profilometry and scanning electron microscopy (SEM). The roughness of both materials increased with etching time; however, lithium disilicate demonstrated a significantly greater response, with Ra values rising from 0.18 µm (control) to 1.26 µm (1 h), while zirconia increased from 0.21 µm to 0.60 µm. ANOVA revealed significant effects depending on the ceramic type, time, and their interaction (*p* < 0.001). SEM images revealed non-selective etching of lithium disilicate, suggesting potential over-etching. The novel acid-etching solution improved surface roughness, especially in lithium disilicate ceramics. An application duration of one hour appears optimal for zirconia, improving surface characteristics while reducing damage; however, further research is required to assess its clinical safety and long-term effects on the mechanical properties of this dental ceramic.

## 1. Introduction

Lithium disilicate was first introduced to the dental field as an indirect restorative material in 1998, marketed as IPS Empress 2 and intended for isostatic pressing manufacture. The latest version, IPS E.max CAD, is offered in a metasilicate state, characterized by 40% lithium metasilicate crystals and a glassy matrix that is bluish in color. The resulting glass–ceramic material after crystallization has the benefit of providing maximum esthetic translucency along with good fracture resistance of ca. 2 MPa and mechanical strength of 360 MPa [1,2].

Zirconia ceramics hold a unique place amongst polycrystalline ceramics due to their excellent mechanical properties. This is a result of their transformation toughening properties and microstructural stability. Proper surface treatment, microstructural control, and understanding of each material’s failure mechanisms are essential for optimizing its durability. As research advanced, zirconia became a viable choice due to the combination of mechanical, esthetic, and biocompatible properties, making it a preferred material for high-stress clinical applications. Yttria-stabilized tetragonal zirconia polycrystal (Y-TZP) ceramics have become a cornerstone in modern restorative dentistry due to their excellent mechanical strength, biocompatibility, and esthetic qualities; 3Y, 4Y, and 5Y zirconia have different translucencies and strengths and are indicated for different clinical scenarios [3,4,5,6].

However, despite the significant improvements in contemporary bonding protocols, achieving durable and reliable bonding to zirconia surfaces remains a challenge, primarily because zirconia is chemically inert and resistant to traditional etching methods used for other ceramics. To improve the bond strength, various surface treatment techniques have been developed, each with distinct mechanisms, applications, and safety profiles [7,8,9,10,11].

Without polishing or glazing, zirconia restorations show a rather low surface roughness after computer-aided design and computer-aided manufacturing (CAD/CAM) machining and sintering. Yahyazadehfar et al. found a mean Ra of ca. 0.19 µm for untreated Y-TZP zirconia [12]. Post-sintering, 4Y-PSZ zirconia shows a similar Ra of ca. 0.22 µm [13]. These numbers fit the oft-used 0.2 µm Ra criterion, below which surface roughness has no effect on biofilm development [14].

Unpolished zirconia’s peak-to-valley roughness (Rz) after sintering ranges from 1.36 to ca. 1.91 µm [12,13]. These values imply that, although with micron-scale grooves from the milling process, as-milled/sintered zirconia shows a rather smooth surface. Surface roughness is greatly influenced by the production process, which consists of milling in a pre-sintered “green” state, followed by roughly 20% shrinkage during firing. Without additional post-finishing treatments, this sintering shrinkage usually smooths the milled surface texture to an Ra value of ca. 0.2 µm [12].

After crystallization, CAD/CAM lithium disilicate materials, such as IPS e.max CAD, show Ra values close to 0.2 µm before polishing or glazing. When processed according to the manufacturer’s protocol, lithium disilicate CAD blocks show a roughness average (Ra) of ca. 0.21 µm, which is similar to the roughness of zirconia. One should note that 0.2 µm Ra is a somewhat substantial threshold; many unpolished ceramic surfaces are located just over this level, suggesting that smoothing would be helpful for reducing biofilm retention. In terms of Rz, lithium disilicate has somewhat higher peak-to-valley heights than zirconia. Compared to ca. 1.9 µm for zirconia, an IPS e.max CAD sample that was milled and crystallized showed an Rz of ca. 2.17 µm. Though the average roughness (Ra) of lithium disilicate is similar to that of zirconia, the surface profile may show somewhat deeper grooves or pits, which would produce a greater Rz. Reflecting the shallow depth of the milling marks, untreated lithium disilicate surfaces show moderate roughness (Rz~2–3 µm) before polishing [13,14].

Sandblasting involves treating the ceramic surface with abrasive particles, typically alumina, to increase surface roughness and create undercuts that facilitate mechanical interlocking with resin cements. This method significantly enhances shear and flexural bond strengths of lithium disilicate and 3Y and 4Y zirconia and induces phase transformation from tetragonal to monoclinic phases by inducing beneficial phase transformations that promote toughening, generating beneficial compressive stresses. Conversely, this may also create microstructural flaws that act as stress concentrators, causing a 20% to 30% additional reduction in strength after several hundred load cycles, leading to earlier failure [15,16,17,18,19].

Contemporary bonding protocols recommend that lithium disilicate restorations should be treated with 50 µm aluminum oxide (Al_2_O_3_) particles (0.1 MPa/1 bar/~15 psi pressure for 5 s at a 5 mm distance and a 45° application angle). For zirconia, the optimal parameters for airborne-particle abrasion (APA) use a pressure of approximately 0.4 MPa, a particle size of 50–100 μm, and a duration of ca. 30 s, leading to increased monoclinic phase volume, which forms a compressive surface layer that resists crack initiation and propagation [20]. Additionally, bonding procedures for dental ceramics often involve acid etching prior to cementation. The primary purpose of acid etching is to increase surface roughness, transforming a flat surface into a more irregular one with microscopic irregularities that enhance the mechanical bonding with dental adhesives and resin cement. Traditionally, acid etching is performed with 4.0–9.5% hydrofluoric acid. The effectiveness of the acid etching varies depending on the type of ceramic and the application time, which ranges from 20 s for lithium disilicate, 30 s for zirconia-reinforced lithium silicate, and 60 s for leucite-reinforced ceramics to 60–120 s for feldspathic porcelain [21,22,23,24,25].

Hydrofluoric acid (HF) chemically interacts with the silica matrix of ceramics, resulting in the formation of volatile silicon tetrafluoride (SiF_4_). Fluorine-containing salts, including lithium fluoride (LiF) and lithium hexafluorosilicate (Li_2_SiF_6_), are produced during the etching process. These salts modify the ceramic surface chemistry by incorporating fluorine residues, hence improving chemical bonding with silane coupling agents. Nevertheless, these leftover salts tend to collect specifically at the ends of needle-like crystals and on the surface, potentially obstructing subsequent bonding processes. Ultrasonic cleaning with water is an efficient technique for eliminating fluoride residues post-HF etching. The method creates small bubbles that implode, generating localized shock waves that remove contaminants, oxidation products, and residual salts from the surface. Surface examination indicates that ultrasonic cleaning markedly diminishes the HF-affected layer and the presence of fluorine-containing compounds, such as silica fluoride salts and other fluorinated residues [26,27,28].

Achieving durable and reliable adhesion between zirconia restorations and dental cements remains a challenge, primarily because zirconia’s inert surface resists conventional etching methods used for silica-based ceramics, such as hydrofluoric acid etching, which is not effective and therefore is not recommended. Recently, a novel etching solution containing HCl, HF, HNO_3_, H_3_PO_4_, and H_2_SO_4_ was developed for zirconia (Zircos-E) and was introduced to the market. The literature suggests that using this 40% HF-based zirconia etching solution provides shear bond strengths (9–11 MPa) comparable to 50 μm Al_2_O_3_ particle airborne abrasion, producing surface irregularities that facilitate bonding; however, there is limited independent data available to evaluate its effectiveness [17,19,27,28,29,30,31,32,33,34,35,36]. This research evaluated the surface roughness of a novel acid-etching treatment for zirconia surfaces, specifically using shorter etching times. If zirconia ceramics demonstrate increased surface roughness (Ra) following the application of this novel acid-etching solution, it may support the recommendation for zirconia etching as a standard step before cementation of zirconia restorations.

## 2. Materials and Methods

The specific aim was to compare the roughness of lithium disilicate and zirconia treated with the novel acid etchant after different application times.

The null hypotheses for the study are the following:

- Prolonged application of the acid etchant significantly increases the surface roughness of zirconia, with longer application times resulting in greater roughness.
- There is a significant difference in surface roughness between zirconia and lithium disilicate ceramics following acid etching.

Chairside CAD/CAM zirconia (ZirCAD, Ivoclar, Schaan, Liechtenstein) and lithium disilicate (E.max CAD, Ivoclar, Schaan, Liechtenstein) blocks, measuring 12 mm in width, 14.5 mm in length, and 18.0 mm in height, were sectioned using a low-speed precision cutter (cutting speed: 8 mm/min) (IsoMet Low Speed Precision Cutter; Buehler, Lake Bluff, IL, USA) to obtain sixty specimens, each with 3 mm thickness, for each ceramic type, resulting in a total of 120 specimens. During the preparation, the values of surface roughness after sectioning and ultrasonic cleaning were compared with the values of the materials after milling (from the literature), and it decided not to finish and polish to create a scenario more representative of the intaglio surface of restorations. The properties of each ceramic material are described in Table 1.

The specimens were divided into 10 groups based on the type of ceramic and the application time of the novel etching material. The application times were 20 s, 60 s, 5 min, 30 min, and 1 h. The group details are presented in Table 2.

To ensure a standardized etching protocol, a micropipette was used to apply the same amount of the etching solution (25 µL per specimen, or approximately 3 drops). After the application time, the specimens were rinsed and air-dried, followed by immersion in an ultrasonic bath (5300 Weep, ultrasonic cleaner, Quala Dental Products, Nashville, TN, USA) containing distilled water and alcohol for 5 min to remove any remnants of the acid used [7,37,38]. The specimens were then placed in a desiccator for one hour, then dry-stored until the surface roughness analysis. The surface roughness of each specimen was then measured. Three-dimensional (3D) images of the texture and topography were captured using a non-contact three-dimensional device (optical profilometer, ZeGage Pro, Zygo, Middlefield, CT, USA). Finally, two-dimensional surface dimensions for each specimen were analyzed using a scanning electron microscope (SEM) (Zeiss Sigma 300 VP-FESEM, Zeiss, Oberkochen, Germany). Details of the composition of the etching solution and the synergistic role of its components are in the Discussion.

## 3. Results

### 3.1. Surface Roughness Measurements

**Figure 1:**
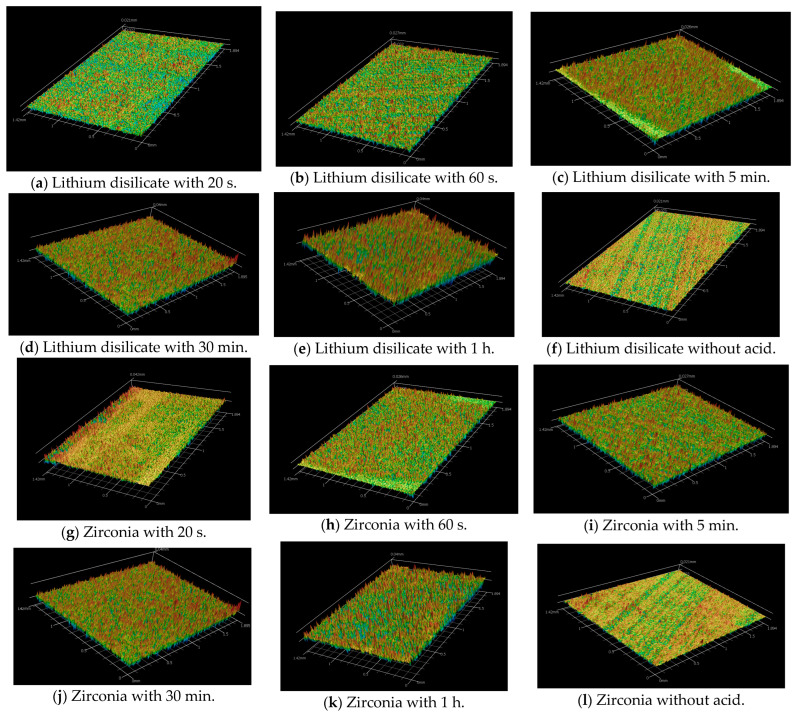
Profilometer images.

**Figure 2:**
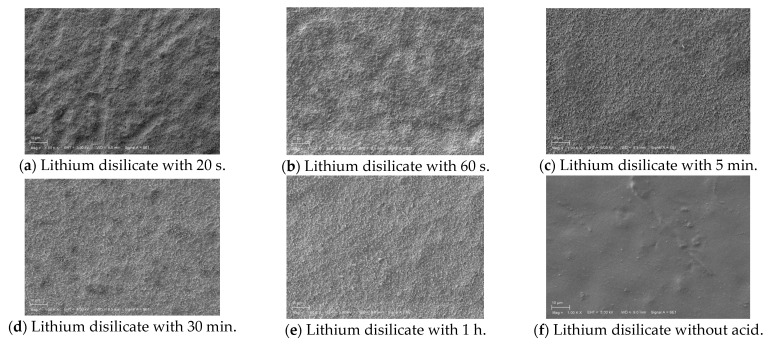

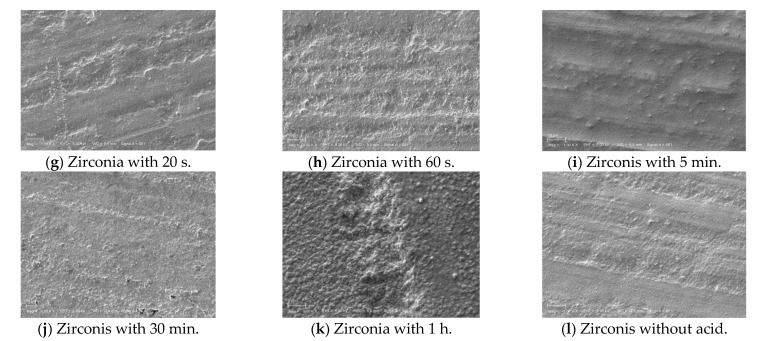
Scanning electron microscope (SEM) images.

The results for surface roughness measurements are included in Table 3 and Figure 1 and Figure 2. Based on the data, there was a clear increase in surface roughness when the alternative treatment was used compared to the control group.

The images of surface roughness can be found in Figure 1 and Figure 2. For the lithium disilicate specimens, the longer the acid contacted the surface, the more roughness was produced. The variables ceramic and etching time were used to conduct a two-way ANOVA. Regarding the variable ceramic, although this research does not intend to rank the materials, there was a notable general difference in surface roughness values between ceramic materials (*p* < 0.001). Lithium disilicate specimens, on average, showed a greater surface roughness than zirconia.

The surface roughness of the specimens was greatly influenced by etching duration (*p* < 0.001). Longer etching times produced notably different surface characteristics compared to shorter times or the control. The variables material and time had a notable interaction for the ceramic and time interaction (*p* < 0.001). This suggests that the ceramic type determined how etching duration affected surface roughness. Practically, lithium disilicate confirmed this interaction effect by exhibiting a far larger increase with extended etching than zirconia. The surface is greatly influenced by both the kind of ceramic and the etching time; LDS reacts more profoundly to prolonged etching with the new etching solution than ZIR (the difference in their behavior over time accounts for the notable interaction). With certain differences in importance, these comparisons show that longer etching times usually raise surface roughness inside each material.

After etching, zirconia usually shows different surface roughness than lithium disilicate; the etching period affects the variation. The control groups for both materials showed significantly reduced surface roughness compared to 30 min, 5 min, and 60 s etching times (*p* < 0.001). Etching for thirty minutes on zirconia produced far more roughness than etching for 20 s, five minutes, and 60 s (*p* < 0.001). Etching for thirty minutes produced noticeably higher roughness than etching for 20 s and 60 s (*p* < 0.001. The control groups and other etching periods within the same material show notable variations. Thus, etching shows a considerable increase in surface roughness over the control—no etching—within both materials. Etching for 30 min produced far more roughness than etching for 20 s, 5 min, and 60 s (*p* < 0.001). Etching for thirty minutes caused noticeably higher roughness than etching for twenty seconds and sixty seconds (*p* < 0.001) for lithium disilicate, but not different from etching for five minutes. These comparisons show that, with specific variations in magnitude, longer etching durations usually raise surface roughness (Table 4).

Table 5 shows the pairwise comparisons for all conditions, highlighting the most significant ones.

For the lithium disilicate (LDS) groups, the pairwise comparisons revealed significant differences between all pairs (*p* < 0.001), except between 5 min vs. 60 s (*p* = 0.028), between 30 min vs. 60 s (*p* = 0.002), and between 30 min vs. 5 min (*p* = 0.002). The LDS specimens etched for 1 h showed the highest surface roughness by far (mean roughness ~1.262) and were notably higher than every other group (*p* < 0.001 for all pairwise comparisons). Specifically, 1 h etching of LDS produced a ~1.08 roughness increase over the unetched control (1.262 versus 0.180; *p* < 0.001) and around a +0.96 roughness difference relative to the shortest 20 s etching (0.305; *p* < 0.001). These variations highlight how much LDS roughness increases with extended acid exposure (ca. 7× its control roughness after 1 h of etching).

Even moderate etching durations resulted in significant roughness increases for lithium disilicate. A 30 min etching (mean roughness of 0.794) produced a +0.614 roughness greater than LDS Co (0.180; *p* < 0.001), and also much greater than a 20 s etching (by ca. 0.489). Though within the control (*p* < 0.001), a 5 min etching (0.612 roughness) nonetheless revealed a +0.432 roughness increase. LDS 60 s (0.439 roughness) even produced a significant increase of ca. 0.259 vs. LDS Co (*p* < 0.001). All etching times ≥1 min resulted in notably rougher LDS surfaces relative to no etching; longer times (30 min, 1 h) had the most noticeable consequences.

Except for ZIR Co vs. ZIR 30 min (*p* = 1.000) and ZIR 30 min vs. ZIR 1 h (*p* = 0.002), the pairwise comparisons for the zirconia (ZIR) groups showed notable differences across all pairings (*p* < 0.001). Though the impact was less noticeable than for LDS, this material also exhibited greater surface roughness with longer etching. For zirconia, notable roughness increases over the ZIR control (mean roughness of 0.211) called for at least 5 min of etching. Treatment for five minutes increased the mean roughness to ca. 0.557, a +0.346 change from the control (*p* < 0.001). Likewise, 30 min (mean 0.564) and 1 h (0.600) etching produced an approximately +0.353 and +0.389 roughness increase versus the control, respectively (both *p* < 0.001). By contrast, short 20 s or 60 s etching of ZIR did not show a statistically significant difference from the unetched surface (e.g., 60 s: 0.353 vs. 0.211, *p* ≈ 0.09). This implies that zirconia needs a longer exposure to the etchant to noticeably raise surface roughness (by around 2.5–3× at 5–60 min, as opposed to no change at 20 s; see 0.600 vs. 0.211 for 1 h vs. control).

Again, although it was not the aim of this research to rank the materials, reflecting the interaction effect, lithium disilicate surfaces became markedly rougher than their zirconia equivalents at longer etching durations. For instance, LDS 1 h produced an approximately 0.662 greater roughness than ZIR 1 h (1.262 vs. 0.600); *p* < 0.001. At 30 min, LDS (0.794) also surpassed ZIR (0.564) by almost +0.230 (*p* < 0.001). Given enough time, these variations are considerable and noteworthy, suggesting that the new etching solution is significantly more aggressive at roughening LDS ceramics than ZIR. In contrast, over shorter times (less than 5 min), there was no noticeable ceramic variation; for example, at 5 min, the average roughness for LDS vs. ZIR (0.612 vs. 0.557) varied by just +0.055 (n.s., *p* = 0.99). In other words, zirconia and lithium disilicate acted similarly with short etching; however, extended etching produced a far higher surface roughness in LDS than in ZIR.

The most notable pairwise variations took place between the extreme settings, specifically, between the etched and control surfaces. Roughly closely behind previous LDS 1 h trials, lithium disilicate with 1 h etching against the control exhibited the greatest effect size (~1.08 roughness increase). The most important differences for zirconia were between 5–60 etching and the control (ca. 0.35–0.39 roughness increase). These findings show, then, that etching time is a key element in improving surface roughness and that lithium disilicate becomes considerably rougher from prolonged etching than zirconia, particularly when treated specimens are compared to their untreated controls. All of the differences were statistically significant at *p* < 0.001 (for both Tukey’s HSD and Bonferroni adjustments), suggesting strong effects in those comparisons.

### 3.2. Profilometer Images

The profilometer observations of the prepared lithium disilicate and zirconia surfaces treated with acid for 20 s, 60 s, 5 min, 30 min, 1 h, and no acid (control group) are shown in Figure 1. The three-dimensional images generally reveal that prolonged acid application results in a progressively rougher surface. The smoothest surface was observed in the control group, which did not receive acid treatment.

### 3.3. Scanning Electron Microscope (SEM) Images

**Figure 3:**
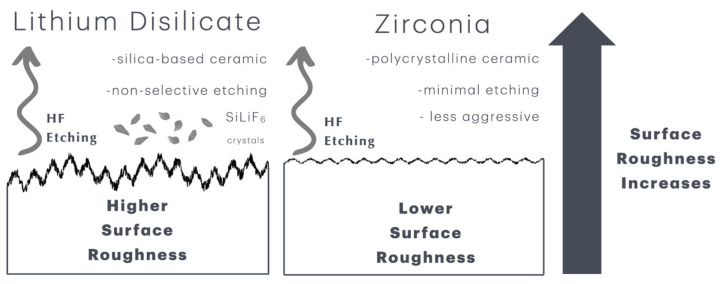
Schematic diagram of the mechanism of action of HF etching on lithium disilicate (**left**) and zirconia (**right**) surfaces. The silica-based composition of lithium disilicate is highly sensitive to the highly concentrated etching solution, producing rougher surfaces but with damage to and substantial loss of structure. The polycrystalline nature of 5Y-TZP zirconia surfaces is more resistant to etching, but the synergistic action of the components of the etching solution produces less aggressive, minimal etching with increased surface roughness.

The SEM observations of the prepared lithium disilicate and zirconia samples treated with acid for 20 s, 60 s, 5 min, 30 min, 1 h, and without acid application are presented in Figure 3. The images clearly show increased microscopic roughness with longer durations of acid application. The control group, which did not undergo acid treatment, exhibits the smoothest surface.

## 4. Discussion

Acid etching prepares the restoration’s intaglio surface for bonding. Proper polishing, delicate handling, avoiding aggressive particle abrasion, using gentler abrasives when needed, and accurate polishing can decrease microcrack initiation and coalescence, preserving ceramic mechanical integrity and service life. To enhance ceramic tooth repair lifespan and function, regular monitoring and clinical treatments are essential [15,35,39].

Research indicates that hydrofluoric acid etching protocols can produce a porous and irregular surface on vitrified Y-TZP ceramics, thereby enhancing micromechanical interlocking with resin cements. The process can yield a surface exhibiting dispersed porous characteristics akin to partially dissolved glass-ceramics, thereby enhancing the bonding potential [9,33].

Etching duration greatly affects dental ceramic wettability and surface roughness. Surface roughness (Ra) increases with etching duration, creating micro- and nano-scale porosities that improve the micromechanical bonding surface area. High-concentration HF (40–48%) for 60 s may improve surface topography and bond strength [18] (Figure 3).

Longer etching times increase ceramic surface roughness and wettability. Short etching times produce small holes, whereas extended ones create uneven grooves and deeper pores. Some modern methods use modest heat or agitation to create effective roughness in 30–60 min, making clinical application more practical [19]. Extended etching (e.g., 160 s) dissolves the matrix around crystals, leading to protruding crystal structures and elongated, randomly oriented lithium disilicate crystals measuring approximately 2.56–2.97 μm [28,35,36,37]. In our study, the etching solution produced non-selective removal of crystals and glass matrix, probably due to the synergistic action of the components (Figure 1b,d,e,f,h,j,l) [29,32].

In the zirconia groups (Figure 2b,d,f,h,j,l), our findings are corroborated by the study on the association of Zircos-E etching and silica coating with different ferrule designs by Alqutaibi et al. [31]. They achieved better results (*p* < 0.001) for endocrown prosthetic designs on molar teeth. Compared to CoJet silica-coated specimens, the Zircos-E etched zirconia specimens showed non-statistical and significantly different behavior. Their results implied that, unlike alumina air particle abrasion, which can embed alumina particles into the zirconia surface and harm it, surface treatments that produce a more homogeneous and microroughened surface, such as Zircos-E etching and silica coating, improve the bonding interface more effectively [31]. However, this could be observed clearly only in the 1 h etching group (ZIR 1 h) (Figure 2j,l).

The literature suggests that 40% HF is effective for achieving significant roughness within 1–2 h, but tests should include shorter durations (15, 30, 60, 120 min). Shortening the etching time makes clinical application more practical, and effective roughness can be achieved in 30–60 min [3,29,38,39].

Increasing the HF etching time results in a higher surface roughness of lithium disilicate ceramics. Specifically, Ra values increase with longer etching durations, indicating a rougher surface. For example, the Ra values reported for no etching were 0.180 ± 0.035 μm and increased progressively to 1.262 ± 0.156 μm for 60 min of etching. SEM micrographs also show more pronounced surface disruptions with longer etching times, such as 5 min, 30 min, and 60 min, compared to shorter durations, such as 20 s [23,40] (Figure 1b,d,f,h,j,l).

The increase in surface roughness of the lithium disilicate specimens did not directly correlate with higher bond strengths. This is attributed to the silica-based composition, which is sensitive to concentrated etching solutions, resulting in rougher surfaces that may incur structural damage and significant loss of material integrity. The aggressive and non-selective behavior of Zircos-E on lithium disilicate surfaces can be attributed to the chemical properties and structural composition of lithium disilicate, along with the strong acidity and broad-spectrum reactivity of the etching solution.

Lithium disilicate (Li_2_Si_2_O_5_) is a glass-ceramic composed of a glassy matrix interspersed with needle-like crystalline phases. According to Poulon-Quintin et al. [24], hydrofluoric acid preferentially attacks the silica-rich glassy phase, leading to selective dissolution and increased surface area. However, Zircos-E, unlike pure HF, contains multiple strong acids (HF, HCl, H_2_SO_4_, HNO_3_, and H_3_PO_4_), which lower the pH dramatically. In contrast to conventional HF etching, which selectively targets the glassy matrix, Zircos-E has a broader attack spectrum due to (1) the action of HNO_3_ (oxidizing agent) and HCl (chloride complexation) as these components can disrupt oxide bonds and increase the permeability of crystalline regions; (2) the role of H_2_SO_4_ (a strong acid and dehydrator), which may alter both glassy and crystal boundaries; and (3) the presence of HF, which remains the primary etchant but is amplified by the surrounding acid environment. This results in nonselective etching, where not only the glassy matrix but also crystalline lithium disilicate needles may undergo degradation or surface damage, especially at prolonged exposure times (e.g., ≥30 min). It is evident that prolonged acid exposure in multi-acid solutions leads to microstructural breakdown and over-etching, which could manifest as increased surface roughness beyond optimal levels and potential loss of micromechanical stability, as needle structures may be eroded rather than just exposed [19,26,27,29].

The efficacy of the Zircos-E acid-etching solution was found to be superior for surface treatment of ultra-translucent zirconia. By significantly altering surface topography and increasing roughness, it does not induce phase change and can sustain bond strength over time, although its initial bond enhancement may be less pronounced than that after sandblasting [19,33,39].

### 4.1. Synergistic Acid Etching of 5Y-TZP Zirconia: Roles of HF, HCl, H_2_SO_4_, HNO_3_, and H_3_PO_4_

Etching 5 mol% yttria-stabilized tetragonal zirconia polycrystal (5Y-TZP) ceramics presents difficulties owing to the chemical inertness and elevated crystalline content of zirconia. Zirconia exhibits resistance to conventional acid etching, in contrast to silica-based ceramics, which are readily etched by hydrofluoric acid (HF). Zircos-E is a multi-acid etching solution utilized in dentistry to chemically roughen the surface of zirconia, thereby enhancing resin bonding. The composition includes hydrofluoric acid (HF), hydrochloric acid (HCl), sulfuric acid (H_2_SO_4_), nitric acid (HNO_3_), and phosphoric acid (H_3_PO_4_). Investigations have examined the effects of this acid combination, applied for durations from seconds to hours, on the surface chemistry and topography of zirconia. This report analyzes the effects of each acid in Zircos-E on 5Y-TZP, emphasizing etching durations ranging from 30 s to 1 h, and any identified “saturation” phenomena in surface changes [19,41].

### 4.2. Rationale for the Multi-Acid Composition (Zircos-E)

Hydrofluoric acid (HF) is the primary etchant in Zircos-E. HF can attack zirconia by forming soluble zirconium fluoride complexes, creating microscopic porosities. In fact, HF alone has been shown to produce notable surface roughening on dental zirconia when used under sufficiently aggressive conditions. Kim et al. [19] observed that zirconia surfaces immersed in HF develop increasing irregularities with longer exposure and higher temperature, though this also induces a tetragonal-to-monoclinic phase transformation. Thus, HF is critical for micromechanical roughening, but its use in isolation may require high concentration, heat, or long duration to be effective and may alter the crystalline phase [19,40,41]. Table 6 summarizes the roles of HF, HCl, H_2_SO_4_, HNO_3_, and H_3_PO_4_ on the synergistic acid etching of 5Y-TZP zirconia.

### 4.3. Effects on Surface Chemistry and Phase Composition

One important aspect of surface chemistry is whether the crystalline phase of zirconia is altered by etching. A well-known concern is the tetragonal-to-monoclinic (t→m) transformation, which can occur if zirconia is thermally or chemically stressed. Pure HF etching has been shown to induce this phase change on the surface [40].

Studies indicate that Zircos-E does not significantly induce t→m transformation under appropriate conditions. Kim et al. [17] demonstrated that strong acid etching with Zircos-E did not induce any detectable phase change in 5Y-TZP, as confirmed by XRD analysis. Although Zircos-E exhibits high acidity, the combination of various acids may facilitate a more regulated etching process, thereby preventing localized stress that could induce phase change. The combination likely etches uniformly at the grain boundaries and in the cubic phase-rich regions rather than causing abrupt lattice disruption. The outcome is a surface that is chemically roughened while remaining structurally intact [40].

Yoshida et al. [43] observed that prolonged HF treatment (especially at elevated temperatures) causes the appearance of the monoclinic phase alongside the tetragonal phase in Y-TZP. This phase transformation is accompanied by volume expansion that can create microcracks, potentially weakening the material.

Multi-acid etching can result in the presence of specific residues or shifts in surface chemistry elements. Etching with HF or HF-containing mixtures frequently leads to the chemisorption of fluoride onto the surface. Although published dental literature contains limited specific X-ray photoelectron spectroscopy (XPS) data on Zircos-E-etched 5Y-TZP, it is plausible that Zr–F or Y–F bonds may form at the outermost layer. It is generally advised to perform thorough rinsing following etching to eliminate loosely bound fluorides or precipitates.

Due to the intense oxidizing atmosphere (HNO_3_), the zirconium surface remains totally oxidized (ZrO_2_). Acids may remove surface impurities and organics, leaving a clean oxide. The acid attack partially disrupts the Zr–O–Zr network, yielding –OH (hydroxyl) terminated surfaces when rinsed. These –OH groups improve silane or MDP primer chemical bonding. After neutralization, Zircos-E etching increases surface oxygen-containing functional groups, enhancing reactivity.

If H_3_PO_4_ deposits phosphate on zirconia, Zr–O–P groups may develop. There is little direct proof; however, some primers can be compared. MDP (10-methacryloyldecyl dihydrogen phosphate) forms surface zirconium phosphate salts that bind firmly. A Zr phosphate layer could be formed by phosphoric acid. This is speculation; most authors do not highlight Zircos-E phosphate residues. A phosphate monolayer reaches a saturation threshold, where more H_3_PO_4_ has a declining effect. A lengthy etching may not boost phosphate uptake indefinitely; a short etching may do so.

In the context of surface chemistry, “saturation” refers to a point during etching where further exposure does not yield additional chemical change. For instance, once the easily accessible grain boundaries and cubic phase regions of 5Y-TZP are etched by the acid, the reaction may slow down, effectively saturating the surface with microporosities and chemical species. Ansari et al. [29] reported that etching 5Y-TZP for 60 min produced pronounced morphological changes (observed on scanning electron microscopy) and significantly improved bonding, whereas the same treatment on 3Y-TZP had a limited effect. This suggests that the 5Y zirconia’s chemistry “saturates” faster—its higher cubic content is more readily etched, achieving maximal surface alteration within ca. 1 h. Beyond that, additional etching time yields little new roughness or bond strength for 5Y (and in 3Y, the acid would have already yielded as much as that structure would allow).

Zircos-E’s combined acids aggressively attack 5Y-TZP’s surface but, if properly controlled, preserve the bulk phase structure (avoiding excessive monoclinic conversion). The surface chemistry reaches an altered state (rich in irregular oxide topography, possibly with some fluorides or phosphates) after tens of minutes of etching, after which a plateau or saturation in chemical change is observed.

### 4.4. Effects on Surface Roughness and Morphology

Surface roughness tends to be higher in air abrasion-treated zirconia compared to etching treatments. However, air abrasion produces irregular and uneven roughness, which can weaken the ceramic structure and cause material loss. In contrast, etching creates more uniform and controlled roughness, which is sufficient to enhance bonding without compromising the integrity of the zirconia surface [42,44].

Sokolowski et al. [30] found that although etching slightly reduced the average roughness parameter (Ra), the differences were statistically insignificant. However, etching significantly reduced the mean spacing of profile irregularities (RSm) (74.04 ± 12.74 μm for the control sample and 50.42 ± 13.62 μm for the etched sample), indicating a denser, more uniform pattern of micro-retentions that could enhance micromechanical bonding. The surface roughness values (Ra) were 0.6160 ± 0.0601 μm for the sandblasted surface (control) and 0.5636 ± 0.0174 μm for the sandblasted and etched surface, with no statistically significant difference between them. Additionally, the roughness parameters were analyzed at 1000×, 5000×, and 15,000× magnification, and significant differences were observed in parameters such as the mean spacing of profile irregularities (RSm). The SEM analysis revealed notable differences in the surface topography of the control and etched specimens. The etched specimens displayed more homogeneous and evenly distributed surface irregularities. Additionally, chemical analysis demonstrated a decrease in surface aluminum concentration post-etching, indicating the effective removal of alumina residues. It was concluded that zirconia surface etching with Zircos-E significantly enhanced surface roughness [30].

Cho et al. [42] applied a 2 h Zircos-E etching protocol, combined with thorough cleaning, annealing, and chemical surface modification, to optimize the bond strength and longevity of zirconia restorations. An annealing process was added to simulate clinical conditions and remove residual surface remnants. Percentage differences in the elements were observed among the groups with different conditioning times. Elements such as fluorine, carbon, nitrogen, and oxygen were observed in the test groups that used distilled water as a rinsing agent after surface conditioning. Surface roughness values after annealing were 0.539, 0.638, and 0.539 in the groups that used the Zircos-E etching system for 1, 2, and 3 h, respectively. The highest Ra values observed after 2 h of application (approximately 0.683) were comparable or superior to the roughness achieved by traditional air abrasion techniques [42,45].

Surface roughness generally correlates positively with bond strength in zirconia surface modifications. Increased roughness enhances mechanical interlocking between the zirconia and resin cement, leading to improved adhesion and bond durability [17,46,47].

The LDS 30 min and LDS 1 h group specimens were significantly rougher than the LDS 20 s and LDS 60 s specimens (*p* < 0.001), but not significantly different from the LDS 5 min specimens, indicating a plateau or saturation in roughening between 5–60 min. In contrast, zirconia showed more modest increases and required longer exposure times to achieve moderate roughness. This suggests not just effectiveness but greater susceptibility to acid degradation, which aligns with the “more aggressive” pattern observed in SEM specimens of the lithium disilicate groups. Poulon-Quintin et al. [24] provided chemical and structural evidence that HF etching of LDS is not strictly selective to the glassy phase since HF attacks both the glass matrix and the Li_2_Si_2_O_5_ crystalline phase, producing SiLi_2_F_6_ precipitates on the surface of the crystals. The formation of plate-like SiLi_2_F_6_ particles contributes to a highly irregular, nano-roughened surface, increasing both mechanical interlocking potential and chemical reactivity. This etching mechanism may lead to non-selective etching, especially at extended exposure durations, unlike the typically selective glass-phase dissolution expected in silica-based ceramics. Thus, the higher roughness observed at longer etching times for LDS can be interpreted as a result of over-etching, damaging not only the matrix but also crystalline structures, leading to extreme surface modification [24].

Zirconia lacks a silica glass phase and is resistant to HF etching. Short HF exposures (e.g., 20 s or 60 s) did not significantly increase surface roughness. Roughness increased significantly only after 5–30 min, suggesting limited etching ability and higher chemical stability, consistent with the polycrystalline, non-silica-based nature. The dramatic surface roughness in LDS is not always beneficial, as excessive roughness from aggressive etching can compromise mechanical integrity and bonding predictability. Poulon-Quintin et al. [24] stressed the importance of controlling etching time and post-etching ultrasonic cleaning to remove fluoride residues and optimize surface chemistry for silane bonding. Therefore, understanding the compositional and structural differences between lithium disilicate and zirconia is essential to define etching protocols that maximize bonding without inducing damage. The structural degradation and formation of fluorinated compounds (e.g., SiLi_2_F_6_) support this hypothesis. This contrasts with zirconia, which remains more chemically stable and less reactive under similar conditions.

Chemical etching with Zircos-E produces notable changes in the surface topography of 5Y-TZP, which in turn influence mechanical interlocking with resin cements. Researchers have evaluated surface roughness (e.g., by SEM imaging or profilometry) for various etching durations in the range from 30 s to 1 h (and beyond).

Zirconia’s microstructure makes grain boundaries and the acid-soluble cubic phase important targets for the first acid attack. Few obvious changes may occur between seconds to minutes, with 5Y-TZP remaining mostly unetched after 30 s in room-temperature acid. Enhanced conditions, such as mild heating or ultrasonic agitation, can make even short etching times effective. Ultrasonic agitation of Zircos-E accelerates ionization; one study found that ultrasound makes protons attack “more easily,” increasing acidity and dissolving zirconia faster. Manufacturers believe 15–45 min of etching (progressive pore development) provide incremental roughening. Thus, 30 s is normally insufficient, while 30 min is often recommended for a significant roughening at room temperature.

Regarding the quantitative roughness (Ra), Cho et al. [42], focusing on a nitric + HF solution, observed via SEM that a 2 h etching produced a rougher surface than 1 h etching, whereas extending etching to 3 h did not further increase roughness. In fact, the 3 h group appeared slightly less rough, suggesting an optimal etching time exists. They attributed the drop at 3 h to possible over-etching or surface damage that could smooth out some microfeatures [19]. From this, we infer a saturation/optimum point around 1–2 h for that acid combination: beyond ~2 h, additional porosity no longer accumulates and may even coalesce or collapse. Importantly, 2 h optimum aligns with the manufacturer’s recommendation for Zircos-E (approximately 2 h at room temperature) for the maximum roughness [48]. Some newer protocols, however, shorten this by using mild heat or agitation so that effective roughness is achieved within 30–60 min instead, to make clinical application more practical.

Etched 5Y-TZP surfaces typically exhibit a matte, frosty appearance to the naked eye, indicating generalized roughening. SEM micrographs show a microporous network of pits and channels. These features result from preferential dissolution along grain interfaces and within the more acid-vulnerable cubic grains. Lee et al. [49] described the acid-etched zirconia surface as an “excellent three-dimensional network” of micro-undercuts. The multi-acid combination was particularly effective at producing a homogeneously rough surface (as opposed to HF alone, which can sometimes create isolated etch pits) [18]. The multi-acid etch essentially blasts the surface at a microscale: by dissolving the superficial grain structure, it increases the surface area and creates micromechanical interlocks for resin infiltration.

The concept of saturation in roughness refers to reaching a plateau in surface texture despite longer etching times. Evidence of this comes from time-course studies. As mentioned, roughness tends to increase up to a point (e.g., 30–60 min), after which additional time yields diminishing returns. In some cases, over-etching can even reduce bond strength—possibly by over-enlarging surface flaws or reducing the stabilizing phase. Kim et al. [19] cautioned that if etching time is excessive, adhesion may be reduced, citing Cho’s finding that a 3 h etching was counterproductive [42]. Thus, there is a sweet spot: sufficient time to create optimal roughness, but not too much that the surface either saturates (no new roughness) or starts to erode in an undesirable way. For practical purposes, many dental studies converge on 30 min to 1 h at ~30 °C as an effective window for Zircos-E etching of 5Y-TZP. Within this window, roughness steadily increases; beyond it, the changes level off. This is the saturation behavior often noted in the context of multi-acid etching.

To visualize the roughness development, manufacturers have provided SEM images: after 15 min of Zircos-E etching, initial fine pits appear; at 30 min, porosity is visibly greater; by 45–60 min, a dense microporous structure covers the surface. Further etching mainly deepens the existing pits rather than creates new ones, which is why the development plateaus. In summary, surface roughness increases rapidly early on and then “saturates” as etching time approaches an hour under typical conditions.

Focusing on in vitro experiments relevant to clinical use, we can summarize how each component of the multi-acid solution or their combination would affect 5Y-TZP. Hydrofluoric acid (HF) has been investigated as a chemical surface treatment for zirconia, either alone or in combination with other acids. Cho et al. [42] evaluated the effect of an etching solution composed of nitric acid and hydrofluoric acid and reported that surface changes and bond strength improvements were dependent on etching time, with optimal results observed at 2 h. Kim et al. [17] compared different etching protocols and noted that multi-acid solutions containing HF were effective in modifying zirconia surfaces and enhancing shear bond strength, while alternative approaches such as a cloud-based etching system showed reduced efficacy. Ansari et al. [29] demonstrated that a multi-acid etching solution, which included HF among other acids, significantly altered the surface morphology of high-translucency zirconia and improved bond strength after 30 min of application. Across these studies, HF plays a critical role in the etching mechanism, but its effectiveness appears to be enhanced when combined with other acids and applied for clinically relevant durations.

The combination of nitric and hydrofluoric acids (HF/HNO_3_) has been explicitly studied by Cho et al. [42] who used a Zircos-E type etchant, describing it as a “nitric acid–hydrofluoric acid compound” applied at room temperature for varying times. They found that 2 h of HF/HNO_3_ etching maximized surface roughness, whereas 1 h was less effective and 3 h could be detrimental. In terms of bond strength, etching with the HF/HNO_3_ solution provided higher shear bond strengths to resin cements than air abrasion or silica coating in some resin systems. This underscores the synergy of HF and HNO_3_: HNO_3_ alone would not etch, but it amplifies HF’s effect such that long exposures rival mechanical roughening. The saturation behavior here was clear—the peak effect at 2 h indicates that HF + HNO_3_ etching had an optimal duration.

When all five acids act together (HF/HCl/H_2_SO_4_/HNO_3_/H_3_PO_4_), studies generally report enhanced surface roughness and good short-term bond strength, particularly on highly translucent zirconia. Ansari et al. [29] tested a commercial multi-acid solution (Zirconia Etching Solution (ZES)) on two zirconia types: 5Y-TZP (high-translucency anterior zirconia) and 3Y-TZP (standard zirconia). After 60 min of etching, SEM showed markedly irregular, rough surfaces for both materials. This result highlights a unique interaction between acid etching and zirconia composition: multi-acid etching is particularly effective on 5Y-TZP (which has more cubic phase). The authors attributed this to the 5Y material’s higher cubic content being more amenable to acid dissolution, whereas 3Y’s tetragonal grains are more acid-resistant.

In essence, the combination of HF/HCl/H_2_SO_4_/HNO_3_/H_3_PO_4_ can selectively exploit the 5Y-TZP’s microstructure, yielding a rougher surface for bonding on 5Y than on 3Y. Ansari et al. [29] also confirmed that this etching (60 min) did not compromise zirconia’s hardness, indicating the bulk mechanical properties were intact. They even concluded that 30 min of etching was sufficient to significantly enhance the bond strength of the 5Y zirconia, aligning with the idea that after ~30 min, the effect on that material saturates.

Despite mixed outcomes, most peer-reviewed dental articles agree that Zircos-E etching creates a distinct, microporous surface that can improve resin cement adhesion, especially for newer 5Y zirconia materials. The combination of acids tends to yield a more uniform roughening than HF alone and does so without the need for abrasive blasting (which can introduce microcracks). When comparing individual acid impacts, HF is indispensable for actual material removal; HCl and HNO_3_ together markedly accelerate etching (neither is sufficient alone); H_2_SO_4_ strengthens the acid environment; and H_3_PO_4_ fine-tunes surface chemistry for bonding. It is the synergy—each component acid amplifying the others—that allows Zircos-E to etch the 5Y-TZP material in clinically reasonable times.

Focusing on etching times between 30 s and 1 h, the following practical implications can be drawn. At 30 s, Zircos-E etching of zirconia is just beginning. There is little evidence of substantial topography change at such a short interval unless extraordinary measures (high heat/ultrasound) are used. Most studies do not even consider 30 s etching due to zirconia’s inertia. A minor initial acid attack might clean the surface and initiate grain boundary erosion, but no significant roughness or porosity is developed yet. In other words, 30 s is below the threshold needed to “activate” the synergistic effects of the acids on 5Y-TZP.

By 5–10 min, slight micro-etching occurs. If the solution is agitated or heated (e.g., some protocols use an ~80 °C water bath for 10 min, as in a “smart etching” approach), measurable changes can start to appear. Kim et al. [17] described smart-etching (sandblasting + acid) for 10 min at 80 °C that slightly increased roughness. Thus, within the first few minutes to a quarter-hour, results vary widely with technique. Generally, short etching (<15 min) at room temperature is insufficient for Zircos-E to fully work on 5Y-TZP.

Around 30 min, the zirconia surface etched with Zircos-E (room temperature, no ultrasound) shows a clearly roughened, matte texture. Roughness values (Ra) start to rise into the “minimally to moderately rough” range (~1 µm or more) as micropores form. Manufacturers often quote 20–30 min as a treatment time when using auxiliary devices (such as ultrasonic baths) to accelerate the process. If one were to etch at a slightly elevated temperature (for example, 40–50 °C), 30 min might achieve a near-optimal surface.

At 1 h (60 min), Zircos-E etching at room temperature without special equipment typically achieves its full effect on 5Y-TZP. The surface is maximally roughened (short of much longer exposure). Studies using ~1 h of etching report significant topographical changes and bond outcomes. For instance, in one experiment, a 1 h etching improved micro-shear bond strength, though slightly less so than a 2 h etching (Kim et al.). Between 30 min and 60 min, there is a continued increase in the density and depth of micropores, but the rate of new feature formation slows down, indicating an approach to the saturation point in roughness. Practically, many authors select 1 h as the etching time for Zircos-E in their protocols, balancing efficacy with efficiency.

It is worth noting that extending to 2 h could further etch the surface slightly (as shown by Cho et al. [42]), but with diminishing returns. Prolonged etching (2–3+ h) risks acid saturation effects such as depletion of active HF (if the acid volume is small relative to surface area) or re-precipitation of reaction products (e.g., fluorides or sulfates) onto the surface, which could passivate further etching. Indeed, the drop in roughness after 3 h suggests that over-etching can lead to a form of saturation or even erosion of the microstructure.

Etching times of 30–60 min are generally optimal for Zircos-E on 5Y-TZP at room temperature. Shorter times (from seconds to a few minutes) are inadequate to harness the synergy of the acids, whereas much longer times yield little additional benefit and may introduce negative effects. The combination of acids accelerates the etching, so that within an hour, a quasi-steady state is reached: the surface is as rough and chemically activated as it needs to be. This is the “saturation” in effect—a plateau in the etching curve. Clinically, this means that a practitioner or laboratory technician would not gain much by etching longer than ~1 h (unless using a weaker solution), and they should also etch at least a few tens of minutes to see a tangible effect.

In conclusion, the synergistic acids in Zircos-E each contribute to etching 5Y-TZP: HF creates the roughness, HCl and HNO_3_ accelerate and deepen the etch, H_2_SO_4_ intensifies it, and H_3_PO_4_ helps optimize surface chemistry. The combination leads to a chemically and mechanically receptive zirconia surface, with an optimal etching time in the tens of minutes, where surface modifications plateau. These insights from dental research provide a roadmap for effectively using multi-acid etchants to improve zirconia bond durability while avoiding over-etching.

It is clear that the synergistic multi-acid etching of 5Y-TZP is a double-edged sword: highly effective in generating a bondable surface (especially within an optimal timeframe) but requiring careful control to avoid plateaus or negative effects from over-etching or residues. When performed correctly, Zircos-E etching enhances micromechanical retention and preserves surface integrity, offering a viable chemical alternative or adjunct to traditional airborne abrasion for achieving durable resin–zirconia bonds.

### 4.5. SEM Analysis

Acid etching creates a uniform microporous surface with tiny grooves, enhancing the surface for bonding without aggressive roughening. These microstructural changes are observable via scanning electron microscopy, which confirms these characteristic surface features [11]. In this study, SEM analysis showed that etching for 60 min altered the morphological characteristics, especially in highly translucent specimens, resulting in a more pronounced surface irregularity [30].

The current evidence indicates that chemical etching with Zircos-E, combined with appropriate mechanical surface modifications, offers the most effective approach to optimize bond strength. The protocol emphasizing chemical etching with Zircos-E combined with mechanical roughening and primer application aligns with the current evidence to maximize resin cement adhesion to zirconia restorations. The surface treatment method that yields the highest shear bond strength to zirconia is the Zircos-E etching solution, as it was found to be the most effective among the evaluated methods, significantly improving SBS [31].

The findings of this study are partially corroborated by another study performing surface examination via SEM that showed distinct variations across the groups [39]. While the control group displayed smooth surfaces with connected grain structures, the etched group exhibited markedly roughened and porous surfaces with evident micro-retentions created by selective dissolution of zirconia grains [39]. In our study, this could only be observed in the ZIR 1 h group.

Sales et al. [43] applied a zirconia-specific etching solution (Zircos-E) for approximately 2 h, according to the manufacturer’s instructions, following mechanical treatment. The chemical conditioning enhances the chemical affinity between zirconia and resin cements. They found that the optimal duration for HF etching to maximize bond strength without degrading zirconia was approximately 10 min at room temperature. Beyond this time, bond strength does not significantly increase and may lead to surface degradation or excessive phase transformation. Prolonged HF etching initially increases the monoclinic phase content in zirconia, with the highest monoclinic phase observed around 160 min of immersion. However, at 320 min, the monoclinic phase content decreases slightly, indicating a complex relationship between etching duration and phase transformation [43].

Chemical etching with agents such as Zircos-E effectively roughens the zirconia surface by increasing the surface area and promoting better interfacial adhesion, leading to higher shear bond strength. Overall, chemical etching tends to produce more consistent and reliable improvements in zirconia surface properties compared to other treatments, such as laser etching, which can be variable and technique-sensitive [31,48]. Another study using SEM analysis demonstrated that 2 h Zircos-E etching created a rougher surface morphology favorable for resin infiltration, comparable to or exceeding the effects of air abrasion. The authors concluded that Zircos-E etching can serve as an alternative or adjunct to air abrasion for optimizing zirconia surface roughness and bond strength [44].

### 4.6. Influence of Moisture

The oral environment’s thermal fluctuations and mechanical stresses are critical for bond durability. Studies demonstrate that surface treatments involving vitrification and HF etching sustain higher bond strengths after aging, indicating superior long-term performance. Conversely, such treatments as sandblasting show a decline in bond strength post-aging, likely due to surface damage and phase transformations that weaken the adhesive interface [9].

Thermocycling (i.e., simulated aging) also plays a significant role in assessing the long-term performance of these bonds. The zirconia etching solution did not significantly improve shear bond strength compared to airborne-particle abrasion. It has been shown that the shear bond strength achieved with zirconia etching solutions is comparable to that obtained with airborne particle abrasion, although the use of the etching solution may be limited in the clinical setting due to its hazardous nature [10].

In their experiment, Nasr et al. [38] fabricated thirty-six zirconia disks of ultra-translucent multilayered Katana™ zirconia, which were sintered and divided into three groups (n = 12 each): a control group without further treatment (C), an airborne particle-abraded group (AB), and an acid-etched group (ZE). The AB group underwent surface roughening with aluminum oxide particles, while the ZE group was immersed in the Zircos-E acid solution for two hours at room temperature, followed by an annealing heat treatment to relieve residual stresses and contaminants [38].

### 4.7. Influence of the Sectioning Technique

Grinding with discs can significantly influence the microstructure of Y-TZP zirconia ceramics, producing distinct micromorphological patterns, including deep scratches and subsurface lateral cracks, which depend on the grit size, applied load, and speed. Larger grit sizes of diamond discs tend to produce greater surface roughness and induce more phase transformation (t→m) in Y-TZP zirconia, which involves a volume expansion of ca. 3–4% and can generate internal stresses within the material, with increased monoclinic phase content observed at larger grit sizes. Conversely, smaller grit sizes result in less surface damage and phase transformation, maintaining better mechanical properties. The extent of phase transformation correlates with the aggressiveness of sectioning, with larger grit sizes and more forceful procedures promoting higher monoclinic phase levels [7,50,51].

Conversely, low-speed grinding with water cooling has a positive impact on the mechanical strength of zirconia ceramics by minimizing surface damage and phase transformation, preserving the material’s integrity, and reducing the risk of inducing microcracks and plastic deformation (ploughing) followed by brittle fracture, which contributes to strength degradation [16,50]. For these reasons, we sectioned the specimens at the slowest speed possible to minimize damage (8 mm/min). In addition, to make it more representative of a clinical scenario, we decided not to use the standard normalization as the standard surface roughness test.

Surface treatments that enhance bonding, such as air abrasion and laser irradiation, must be balanced against their potential to weaken the zirconia structure. Microstructural changes such as phase transformation and microcrack formation can compromise long-term durability, especially under cyclic loading conditions in the oral environment [11,50,51].

Air abrasion significantly influences the phase transformation and strength of 3Y-TZP zirconia. It promotes the transformation of the tetragonal phase to the monoclinic phase, which can enhance surface roughness and bonding, but may also induce surface flaws and microcracks, potentially weakening the material [5,11,16,51].

From a clinical perspective, the association between airborne abrasion and etching offers a promising balance by providing durable bonds with minimal risk of phase transformation. Sandblasting remains effective for initial adhesion, but may compromise long-term stability due to phase changes and surface flaws. Acid etching, while less aggressive, offers a chemically stable surface that can sustain bond strength over time, especially when combined with proper resin cements. Nonetheless, reproducing complex oral forces in laboratory settings remains challenging, and further research is needed to optimize protocols for clinical longevity [10,19,23,43,47,50,51].

Future research is suggested on the combination of chemical bonding strategies and mechanical roughening, along with the use of resin cements, which generally yields the most reliable and durable bonds. Ongoing research continues to refine these techniques, aiming to optimize both bond strength and safety for long-term success.

## 5. Conclusions

The alternative etching method effectively increased the surface roughness of the zirconia and lithium disilicate specimens. While acknowledging the role of chemical adhesion in bonding porcelain to zirconia, it can be concluded that etching primarily enhances mechanical retention. This highlights the critical balance needed in surface treatments, as over-etching could negatively impact zirconia strength and structural integrity. The plateau in roughness values and minimal damage observed under SEM suggest that a 1 h treatment can be recommended as optimal, providing enhanced surface roughness for zirconia surfaces without the adverse effects observed at longer etching durations. The novel zirconia etching solution provided non-selective etching of lithium disilicate surfaces, probably due to the synergistic action of the components.

## Figures and Tables

**Table 1:** Ceramic materials used, categorized by their microstructure, physical characteristics, and applications, where σ, Ra, KIc, E, and CTE represent flexural strength, surface roughness, fracture toughness, elastic modulus, and coefficient of thermal expansion, respectively.

Material	Commercial Brand	Crystalline Microstructure	Mechanical Properties *** CTE	Clinical Indication ***
Glass–ceramic Lithium disilicate 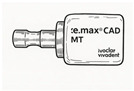	IPS e.max CAD^®^ (Ivoclar)	Needle–like crystals (approx. 70 vol%) Composition: Li_2_Si_2_O_5_ Size: 3–6 µm (length)	σ: 350–450 MPa Ra: 0.21 µm ** KIc: 0.8–1.5 MPa·m^1/2^ E: ~70 GPa CTE: 10.2 ± 0.4 × 10^−6^ K^−1^ (100–400 °C), 10.6 ± 0.35 10^−6^ K^−1^ (100–500 °C)	Crowns, veneers, occlusal veneers (tabletops) ≥ 1.0 mm, inlays, onlays, partial crowns, 3-unit bridges in the anterior and posterior regions (2nd premolar as the terminal abutment) Hybrid abutments in the anterior and posterior region as a single-tooth restoration, hybrid abutment crowns in the anterior and posterior regions
Zirconia 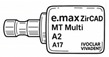	e.max ZirCAD^®^ (Ivoclar)	Homogeneous fine Y_2_O_3_: 4.0–6.0% ZrO_2_: 87.0–95.0% HfO_2_: 1.0–5.0% Al_2_O_3_: 0.0–1.0% Other oxides: <0.2%	σ: ≥ 900 MPa Ra: 0.22 µm * KIc: 5.14 ± 0.07 MPa·m^1/2^ E: 70 ± 2 GPa CTE: 10.6 ± 0.1 10^−6^ K^−1^ (100–400 °C)	Full-contour crowns, 3-unit bridges, and 4- and multi-unit bridges with max. 2 pontics, crown copings, 3-unit and multi-unit bridge frameworks with max. 2 pontics

Note: * [12], ** [13,14], *** information from the manufacturer.

**Table 2:** Groups formed by the ceramic materials and etching time.

Zirconia (ZirCAD, Ivoclar)		Lithium Disilicate (E.max CAD, Ivoclar)		
n	Name/symbol	n	Name/symbol	Etching time
10	ZIR 20 s	10	LDS 20 s	20 s
10	ZIR 60 s	10	LDS 60 s	60 s
10	ZIR 5 min	10	LDS 5 min	5 min
10	ZIR 30 min	10	LDS 30 min	30 min
10	ZIR 1 h	10	LDS 1 h	1 h
10	ZIR Co *	10	LDS Co*	0 (control)

* Groups 6 and F are control groups without any surface treatment.

**Table 3:** ANOVA for the variables CERAMIC (lithium disilicate or zirconia) and TIME (1 h; 20 s; 30 min; 5 min; 60 s).

Cases	Sum of Squares	df	Mean Square	F	*p*
Ceramic	0.758	1	0.758	72.931	<0.001
Time	7.106	5	1.421	136.692	<0.001
Ceramic time	1.768	5	0.354	34.016	<0.001
Residuals	1.123	108	0.010		

Note: type III sum of squares; df: degrees of freedom.

**Table 4:** Descriptive statistics: mean, standard deviation (SD), standard error (SE), and coefficient of variation of surface roughness (Ra) for the lithium disilicate (LDS) and zirconia (ZIR) specimens treated with a zirconia etching solution for different etching times: control (without etching); 20 s; 60 s; 5 min; 30 min; and 1 h.

Ceramic	Time	N	Mean	SD	SE	Coefficient of Variation
LDS	(0) Control	10	0.180	0.035	0.011	0.192
	20 s	10	0.305	0.052	0.017	0.172
	60 s	10	0.439	0.043	0.013	0.097
	5 min	10	0.612	0.096	0.030	0.157
	30 min	10	0.794	0.263	0.083	0.332
	1 h	10	1.262	0.156	0.049	0.124
ZIR	(0) Control	10	0.211	0.047	0.015	0.224
	20 s	10	0.353	0.044	0.014	0.124
	60 s	10	0.353	0.044	0.014	0.124
	5 min	10	0.557	0.066	0.021	0.119
	30 min	10	0.564	0.048	0.015	0.085
	1 h	10	0.600	0.055	0.017	0.092

**Table 5:** Post hoc comparisons for pairwise comparisons of ceramic versus time. Only the relevant pairwise comparisons are exhibited.

		Mean Difference	t	Tukey’s *p*	Bonf. ** *p*
LDS 1 h	LDS 30 min	0.468	10.267	<0.001	<0.001
	LDS 5 min	0.651	14.267	<0.001	<0.001
	LDS 60 s	0.824	18.061	<0.001	<0.001
	LDS 20 s	0.957	20.991	<0.001	<0.001
	LDS Co	1.082	23.721	<0.001	<0.001
ZIR 1 h	ZIR Co	1.051	23.057	<0.001	<0.001
	ZIR 30 min	0.036	0.783	1.000	1.000
	ZIR 5 min	0.043	0.939	0.999	1.000
	ZIR 60 s	0.246	5.403	<0.001	<0.001
	ZIR 20 s	0.246	5.403	<0.001	<0.001
LDS 20 s	LDS 30 min	−0.489	−10.724	<0.001	<0.001
	LDS 5 min	−0.307	−6.724	<0.001	<0.001
	LDS 60 s	−0.134	−2.930	0.145	0.273
	LDS Co	0.125	2.730	0.226	0.488
ZIR 20 s	ZIR 30 min	−0.211	−4.621	<0.001	<0.001
	ZIR 5 min	−0.204	−4.465	0.001	0.001
	ZIR 60 s	−3.678 × 10^−16^	−8.065 × 10^−15^	1.000	1.000
	ZIR Co	0.142	3.125	0.090	0.151
LDS 30 min	LDS 5 min	0.182	4.000	0.006	0.008
	LDS 60 s	0.355	7.794	<0.001	<0.001
	LDS Co	0.613	13.454	<0.001	<0.001
ZIR 30 min	ZIR 5 min	0.007	0.156	1.000	1.000
	ZIR 60 s	0.211	4.621	<0.001	<0.001
	ZIR Co	0.353	7.745	<0.001	<0.001
LDS 5 min	LDS 60 s	0.173	3.794	0.012	0.016
	LDS Co	0.431	9.454	<0.001	<0.001
ZIR 5 min	ZIR 60 s	0.204	4.465	0.001	0.001
LDS 60 s	LDS Co	0.258	5.660	<0.001	<0.001
ZIR 60 s	ZIR Co	0.142	3.125	0.090	0.151
LDS Co	ZIR Co	−0.030	−0.664	1.000	1.000

Note: Standard Error is 0.046 for all pairwise comparisons; ** Bonferroni’s post hoc test.

**Table 6:** Synergistic acid etching of 5Y-TZP zirconia: roles of HF, HCl, H_2_SO_4_, HNO_3_, and H_3_PO_4_.

Acid	Function in Zircos-E	References
**Hydrochloric acid (HCl)**	Maintains low pH; facilitates chloride complexation; assists in superficial oxide removal	D’Alessandro et al., 2024 [18]; Ansari et al., 2018 [29]
**Nitric acid (HNO** **_3_)**	Oxidizes the zirconia surface; enhances the HF action; contributes to optimized etching duration	Kim et al., 2024 [19]; Cho et al., 2017 [42]
**Sulfuric acid (H** **_2_SO** **_4_)**	Increases solution acidity; removes surface-bound moisture; accelerates etching reactions	D’Alessandro et al., 2024 [18]; Sadid-Zadeh et al., 2021 [10]
**Phosphoric acid (H** **_3_PO** **_4_)**	May modify surface chemistry; introduces phosphate groups favorable for bonding interactions	Poulon-Quintin et al., 2021 [24]; Ansari et al., 2018 [29]

## Data Availability

The original contributions presented in this study are included in the article. Further inquiries can be directed to the corresponding author.
